# Endovascular treatment in patients with acute ischemic stroke presenting beyond 6 h after symptom onset: An international multicenter cohort study of the EVA-TRISP collaboration

**DOI:** 10.1177/23969873241277437

**Published:** 2024-09-08

**Authors:** Nabila Wali, Lotte J Stolze, Leon A. Rinkel, Mirjam R Heldner, Madlaine Müller, Marcel Arnold, Pasquale Mordasini, Jan Gralla, Philipp Baumgartner, Corinne Inauen, Laura P Westphal, Susanne Wegener, Patrik Michel, Simon Trüssel, Laura Mannismäki, Nicolas Martinez-Majander, Sami Curtze, Georg Kägi, Livio Picchetto, Maria Luisa Dell’Acqua, Guido Bigliardi, Christoph Riegler, Christian H Nolte, Miguel Serôdio, Miguel Miranda, João Pedro Marto, Andrea Zini, Stefano Forlivesi, Luana Gentile, Carlo W Cereda, Alessandro Pezzini, Ronen R Leker, Asaf Honig, Ivana Berisavac, Visnja Padjen, Marialuisa Zedde, Laurien S Kuhrij, Renske M Van den Berg-Vos, Stefan T Engelter, Henrik Gensicke, Paul J Nederkoorn

**Affiliations:** 1 Department of Neurology, Amsterdam UMC, University of Amsterdam, Amsterdam, The Netherlands; 2 Department of Radiology, Foothills Medical Centre, University of Calgary, Calgary, AB, Canada; 3 Department of Neurology, Inselspital, Bern University Hospital and University of Bern, Switzerland; 4 Institute for Diagnostic and Interventional Neuroradiology, Inselspital, Bern University Hospital, and University of Bern, Bern, Switzerland; 5 Department of Radiology, Kantonsspital St. Gallen, St. Gallen, Switzerland; 6 Department of Neurology, University Hospital Zurich and University of Zurich, Zurich, Switzerland; 7 Stroke Center, Neurology Service, Lausanne University Hospital and University of Lausanne, Lausanne, Switzerland; 8 Neurology and Neurorehabilitation, University Department of Geriatric Medicine FELIX PLATTER, University of Basel, Basel, Switzerland; 9 Stroke Center and Department of Neurology, University Hospital Basel and University of Basel, Basel, Switzerland; 10 Neurology, University of Helsinki and Helsinki University Hospital, Helsinki, Finland; 11 Department of Neurology, Kantonsspital St. Gallen, St. Gallen, Switzerland; 12 Stroke Unit, Department of Neuroscience, Ospedale Civile di Baggiovara, Modena University Hospital, Modena, Italy; 13 Department of Neurology with experimental Neurology, Charité – Universitätsmedizin Berlin, Corporate member of Freie Universität Berlin and Humboldt Universität zu Berlin, and Berlin Institute of Health (BIH), Berlin, Germany; 14 Center for Stroke Research Berlin (CSB), Charité – Universitätsmedizin Berlin, Germany; 15 Department of Neurology, Hospital de Egas Moniz, Centro Hospitalar Lisboa Ocidental, Lisbon, Portugal; 16 Department of Neurology, Hospital de Cascais Dr. José de Almeida, Cascais, Portugal; 17 IRCCS Istituto delle Scienze Neurologiche di Bologna, Department of Neurology and Stroke Center, Maggiore Hospital, Bologna, Italy; 18 Stroke Center and Department of Neurology, Neurocenter of Southern Switzerland, EOC, Lugano, Switzerland; 19 Department of Medicine and Surgery, University of Parma, Parma, Italy; 20 Stroke Care Program, Department of Emergency, Parma University Hospital, Parma, Italy; 21 Department of Neurology, Hadassah-Hebrew University Medical Center, Jerusalem, Israel; 22 Neurology Clinic, Clinical Centre of Serbia, Faculty of Medicine, University of Belgrade, Belgrade, Serbia; 23 Neurology Unit-Stroke Unit, Azienda Unità Sanitaria Locale-IRCCS di Reggio Emilia, Reggio Emilia, Italy; 24 Department of Biomedical Data Sciences, Leiden University Medical Center, Leiden, The Netherlands; 25 Department of Neurology, OLVG, Amsterdam, The Netherlands

**Keywords:** Acute ischemic stroke, endovascular thrombectomy, extended time window

## Abstract

**Introduction:**

After positive findings in clinical trials the time window for endovascular thrombectomy (EVT) for patients with an acute ischemic stroke has been expanded up to 24 h from symptom onset or last seen well (LSW). We aimed to compare EVT patients’ characteristics and outcomes in the early versus extended time window and to compare outcomes with the DAWN and DEFUSE 3 trial results.

**Patients and methods:**

Consecutive EVT patients from 16 mostly European comprehensive stroke centers from the EVA-TRISP cohort were included. We compared rates of 90-day good functional outcomes (Modified Rankin Scale 0–2), symptomatic intracranial hemorrhage (sICH), and 90-day mortality between patients treated in the early (<6 h after onset or LSW) versus extended (6–24 h after onset or LSW) time windows.

**Results:**

We included 9313 patients, of which 6876 were treated in the early and 2437 in the extended time window. National Institutes of Health Stroke Scale (NIHSS) score at presentation was lower in patients treated in the extended time window (median 13 [IQR 7–18] vs 15 [IQR 9–19], *p* < 0.001). The percentage of patients with good functional outcome was slightly lower in the extended time window (37.4% vs 42.2%, *p* < 0.001). However, rates of successful recanalization, sICH, and mortality were similar. Good functional outcome rates after EVT were slightly lower for patients in the extended window in the EVA-TRISP cohort as compared to DAWN and DEFUSE 3.

**Discussion and conclusion:**

According to this large multicenter cohort study reflecting daily clinical practice, EVT use in the extended time window appears safe and effective.

## Graphical abstract

**Graphical abstract:**
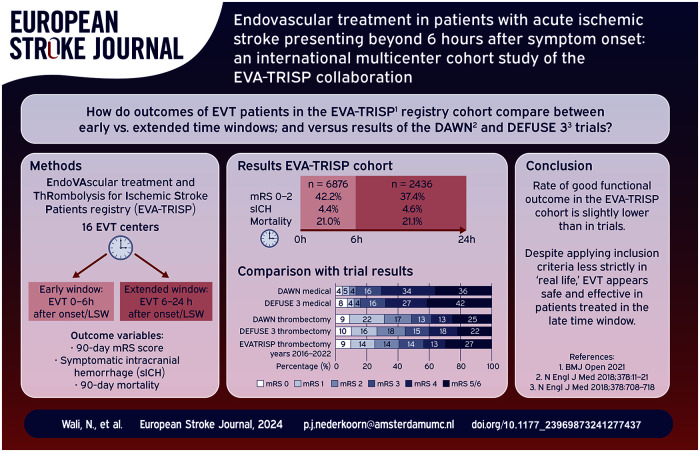

## Introduction

Endovascular thrombectomy (EVT) is a proven effective treatment for acute ischemic stroke (AIS) in patients with a large vessel occlusion.^1–5^ Treatment with EVT up to 6 h after onset or last seen well (LSW) of stroke symptoms is widely implemented as a standard treatment option for AIS. Over the past years the effectiveness of EVT outside of this conventional time window has been further explored and has been proven in randomized controlled trials. The DAWN (DWI or CTP Assessment with Clinical Mismatch in the Triage of Wake-Up and Late Presenting Strokes Undergoing Neurointervention with Trevo) and DEFUSE 3 (Endovascular Therapy Following Imaging Evaluation for Ischemic Stroke) trials have shown the efficacy of EVT in an extended time window up to 16 and 24 h after stroke onset.^6,7^ Following these findings, treatment with EVT in the extended time window has been implemented, enabling AIS patients to be eligible for EVT even if they present beyond 6 h after onset of symptoms if imaging criteria are met.^8,9^ While clinical trials have shown the effectiveness of reperfusion treatment after 6 h from stroke onset or LSW, there are still limited data on the implementation and the effectiveness of reperfusion therapy in extended time windows outside of a trial setting, in daily clinical practice. Previous relatively small cohorts^10–17^ as well as a few more recent and larger real-world cohort studies^18–22^ have evaluated the effects and safety of treatment with EVT beyond 6 h and have shown positive results. In the EndoVAscular treatment and ThRombolysis for Ischemic Stroke Patients (EVA-TRISP) collaboration, our aims were to (1) investigate the proportions of patients treated in the extended time window with EVT over the years (2) compare the patient characteristics and outcomes after EVT in early and extended time windows with real-world prospective data from a large multicenter cohort (3) and to compare real-world outcomes with those observed in the DAWN and DEFUSE 3 clinical trials.

## Methods

### Patient selection

EVA-TRISP is an international multicenter cohort study including mostly European centers in which all consecutive patients receiving EVT are enrolled. Detailed information about the EVA-TRISP study protocol has previously been published.^23^ In short, predefined variables such as patient characteristics, prehospital information, baseline imaging findings, treatment times, EVT procedural information and outcomes after EVT are prospectively collected in 20 comprehensive stroke centers from 10 different countries.

In the current study, 16 EVA-TRISP centers participated. The beginning (and ending) of data gathering varied among the stroke centers (Supplemental File 1), therefore, to account for this discrepancy, we included only data collected from 2016 and onward. Patients were included in analysis if the following inclusion criteria were met; treatment with EVT in the years 2016–2022, an age of 18 years or older, and intracranial occlusion of the internal carotid artery or the proximal middle cerebral artery (M1 and/or M2 segment occlusions). Patients were considered to have been treated in the early time window if they presented with an onset -or LSW- to groin time between 0 and 6 h and considered to have been treated in the extended time window if onset -or LSW- to groin time was between 6 and 24 h. Exclusion criteria were treatment beyond 24 h after onset or LSW or when the treatment window could not be determined due to missing onset, LSW, or groin time.

### Outcomes

Our outcome measures were good functional outcome at 3 months after stroke onset defined as a Modified Rankin Scale (mRS) score between 0 and 2, mortality within 3 months after stroke onset, and the occurrence of a symptomatic intracranial hemorrhage (sICH) according to the ECASS II (European Co-operative Acute Stroke Study II) criteria; which defines sICH as blood at any site in the brain on CT-scan, any documentation of clinical deterioration, or adverse events indicating clinical worsening, or causing an increase in the National Institutes of Health Stroke Scale (NIHSS) score of ⩾4 points.^24^

### Statistical analysis

Statistical analyses were performed with SPSS Statistics version 25 (IBM Corporation, Armonk, NY, USA).^25^ We compared characteristics and outcomes for patients treated with EVT in the early versus the extended time window. Baseline characteristics were compared using the χ2 test to assess differences for categorical variables, and the Mann–Whitney *U* test for continuous variables. Multivariable logistic regression random effect models were used to compare 90-day good functional outcome, 90-day mortality and the occurrence of sICH between patients treated in the early versus the extended time window. Odds ratios and 95% confidence intervals were calculated. In the random effect models we adjusted for age, sex, NIHSS score at presentation, a medical history of atrial fibrillation, hypertension and hypercholesterolemia, treatment with intravenous thrombolysis (IVT) and year of stroke onset, as potential confounders. These potential confounders were selected based on a significant difference (*p* < 0.05) between groups in the baseline analysis. The treatment center was used as a random effects variable, to account for heterogeneity.

### Comparison with trials

Because of the widespread implementation of treatment in the extended time window since the positive outcomes of large clinical trials^6,7^ were published, we also specifically compared outcomes between the early and extended time windows for patients treated between the years 2019–2022. And since the clinical trials excluded patients with a pre-mRS score above 2, we also did additional analyses in which we compared outcomes in early and extended time windows only for patients with a pre-mRS score between 0 and 2.

Furthermore, we explored to which extent the distribution of 90-day mRS scores in the extended time window in this EVA-TRISP real-world study were comparable with mRS scores of patients treated in the intervention group in DAWN and DEFUSE 3.^6,7^ In EVA-TRISP and DAWN the extended time window was defined as an onset or LSW to groin time between 6 and 24 h and in DEFUSE 3 between 6 and 16 h. To be able to compare outcomes fairly we aimed to align our inclusion criteria closely with those of the DAWN and DEFUSE 3 trials, however due to limitations in the database, inclusion criteria could not be matched on imaging criteria, that is, CTA/CTP or MRI results.

### Ethical approval

Approval from local authorities and/or ethical committees has been secured by each treatment center in compliance with national and local regulations.

## Results

### Baseline characteristics

**Figure 1.**
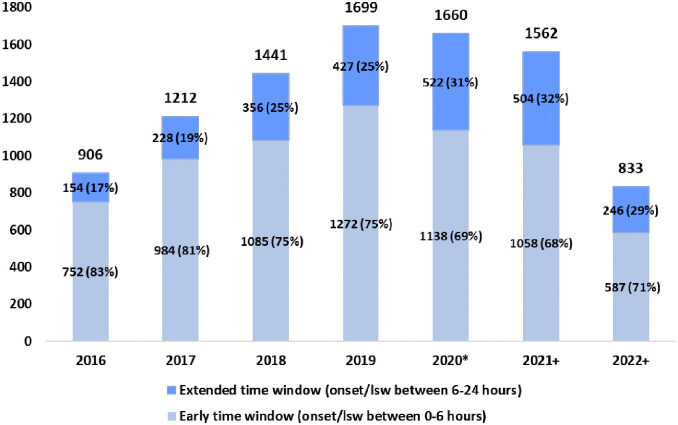
Trend in numbers and percentage of patients treated with EVT per treatment window between 2016 and 2022. *In the beginning of 2020 covid-19 spread widely in the areas where the included centers are located + For some hospitals data for 2021/2022 is missing or incomplete (see Supplemental File).

Between 2013 and 2023, 13,462 consecutive EVT patients were included in the EVA-TRISP cohort. After exclusion of 3144 patients due to not meeting our predefined inclusion criteria, and 1005 patients due to treatment with EVT beyond 24 h after onset or LSW or missing time variables, 9313 patients were available for analyses. See Supplemental File 2 for a summary of numbers and reasons for exclusion of patients in analyses. Of the included patients, 6876 were treated in the early time window and 2437 in the extended time window. Between 2016 and 2022 the proportion of patients treated in the extended time window increased from 17.0% in 2016 to 32.3% in 2021 (Figure 1). In our cohort 29.5% were treated in the extended time window in 2022. However, data collection was not complete for a few centers in this year (Supplemental File 1).

Baseline characteristics for patients treated in the early versus extended time window are shown in Table 1. The median onset or LSW to groin time was 185 min (IQR 143–245) for patients treated in the early time window and 630 min (IQR 460–851) in the extended time window.

**Table 1.** Baseline characteristics per time window.

	Early time window	Extended time window	*p*-Value
Number of patients	6876	2437	
Age, mean (SD)	72.6 (13.5)	72.6 (13.5)	0.893
Missing, *n* (%)	0	0	
Female sex, *n* (%)	3366 (49.0)	1298 (53.3)	**<0.001**
Missing, *n* (%)	4 (0.1)	3 (0.1)	
Medical history, *n* (%)			
Hypertension	4604 (67.0)	1705 (70.0)	**0.007**
Diabetes mellitus	1283 (18.7)	475 (19.5)	0.368
Atrial fibrillation	2588 (37.6)	865 (35.5)	0.052
Hypercholesterolemia	3237 (47.1)	1248 (51.2)	**<0.001**
Current smoking (or stopped <2 years ago)	1265 (18.4)	472 (19.4)	0.240
Coronary heart disease	1131 (16.4)	396 (16.2)	0.668
Prior stroke	904 (13.1)	313 (12.8)	0.872
Independent pre-stroke^a^, *n* (%)	5826 (84.7)	2076 (85.2)	0.148
Not independent pre-stroke^b^, *n* (%)	610 (8.9)	244 (10.0)	
Missing, *n* (%)	440 (6.4)	117 (4.8)	
Transferred from another hospital, *n* (%)	2607 (37.9)	975 (40.0)	**0.044**
Missing, *n* (%)	199 (2.9)	82 (3.4)	
NIHSS at presentation, median (IQR)	15 (9–19)	13 (7–18)	**<0.001**
Missing, *n* (%)	154 (2.2)	38 (1.2)	
Treatment with IVT, *n* (%)	4174 (60.7)	524 (21.5)	**<0.001**
Missing, *n* (%)	0	0	
Successful recanalization on DSA,^c^ *n* (%)	4056 (59.0)	1453 (59.6)	0.136
Missing, *n* (%)	1711 (24.9)	625 (25.6)	
Onset/LSW to groin time, median (IQR)	185 (143–245)	630 (460–851)	**<0.001**
Missing, *n* (%)	0	0	

SD: standard deviation; NIHSS: National Institute of Health Stroke Scale; IQR: interquartile range; IVT: intravenous thrombolysis; DSA: digital subtraction angiography; LSW: last seen well. *p*-values <0.05 were considered significant and are highlighted in bold.

a Defined as a modified Rankin scale score = 0–2.

b Defined as a modified Rankin scale score = 3–5.

c Defined as a modified thrombolysis in cerebral infarction score = 2b − 3.

Patients treated in the extended time window were more often female (1298 [53.3%] vs 3366 [49.0%], *p* ⩽ 0.001), had a lower prevalence of atrial fibrillation (865 [35.5%] vs 2588 [37.6%], *p* = 0.052) but higher prevalence of hypertension (1705 [70.0%] vs 4604 [67.0%], *p* = 0.007) and hypercholesterolemia (1248 [51.2%] vs 3237 [47.1%], *p* =< 0.001) in their medical history. NIHSS score at presentation was lower in patients treated in the extended time window (median 13 [IQR 7–18] vs median 15 [IQR 9–19], p < 0001). Mean age and pre-stroke independence did not differ significantly between the groups. In both time windows a notable proportion of patients were not independent prior to the stroke (defined as a pre-mRS score between 3 and 5): 10.0% of patients treated in the extended and 8.9% in the early time window.

### Outcomes

As seen in Table 1, successful recanalization on digital subtraction angiography (DSA) was achieved in both groups to a similar extent (1453 [59.6%) vs 4056 [59.0%], *p* = 0.136). There was a considerably smaller shift to lower NIHSS scores 24 h after EVT in patients treated in the extended time window (∆NIHSS mean (SD) −1.7 [7.2] vs −3.6 [8.1], *p* ⩽ 0.001). Lower proportion of patients treated in the extended time window had a good functional outcome 3 months after stroke onset (911 [37.4%] vs 2900 [42.2%], *p* < 0.001) (Table 2).

**Table 2.** Outcomes after EVT per treatment window for the years 2016–2022 and the years 2019–2022.

	Years 2016–2022			Years 2019–2022		
	Early time window	Extended time window	*p*-Value	Early time window	Extended time window	*p*-Value
Number of patients	6876	2437		4055	1699	
NIHSS after 24 h, median (IQR)	8 (3–16)	10 (4–16)	**<0.001**	8 (3–16)	9 (4–16)	**<0.001**
Missing, *n* (%)	1078 (15.7)	280 (11.5)		597 (14.7)	219 (12.9)	
∆ NIHSS, mean (SD)	−3.6 (8.1)	−1.7 (7.2)	**<0.001**	−3.7 (7.9)	−1.8 (7.4)	**<0.001**
Missing, *n* (%)	1115 (16.2)	292 (12.0)		615 (15.2)	226 (13.3)	
Symptomatic ICH^a^, *n* (%)	301 (4.4)	113 (4.6)	0.660	169 (4.2)	72 (4.2)	0.775
Missing, *n* (%)	1588 (23.1)	356 (14.6)		825 (20.3)	268 (15.8)	
mRS score after 3 months, (%)			**<0.001**			**<0.001**
0	12.1	8.8		12.3	9.5	
1	15.8	14.3		16.0	14.8	
2	14.2	14.2		13.2	14.0	
3	13.2	14.1		12.3	13.2	
4	10.8	13.2		10.7	12.4	
5	4.4	5.6		4.2	5.5	
6	21.0	21.1		20.2	21.3	
Missing, *n* (%)	8.4	8.6		10.2	9.3	
Good functional outcome^b^, *n* (%)	2900 (42.2)	911 (37.4)	**<0.001**	1718 (42.4)	650 (38.3)	**<0.001**
Mortality^c^, *n* (%)	1442 (21.0)	514 (21.1)	0.853	821 (20.2)	362 (21.3)	0.457

NIHSS: National Institutes of Health Stroke Scale; IQR: interquartile range; SD: standard deviation; mRS: modified Rankin scale.

∆ NIHSS after 24 h minus NIHSS at presentation. *p*-values <0.05 were considered significant and are highlighted in bold.

a According to ECASS II criteria.

b Defined as a modified Rankin scale score = 0–2, 3 months after stroke onset.

c Defined as a mRS score = 6, 3 months after stroke onset.

In the adjusted logistic regression analysis (Table 3), treatment in the extended time window was associated with lower odds of good functional outcome (adjOR 0.82 [95% confidence interval (CI) [0.73–0.93], *p* = 0.002). The occurrence of sICH and mortality at 3 months were similar between the early and extended time window (Table 3).

**Table 3.** Results of univariate and multivariable random effect binomial regression analysis of outcomes after EVT in extended versus early time window.

	Unadjusted OR	*p*-Value	Adjusted OR	*p*-Value
Symptomatic ICH^a^	0.95 (0.76–1.19)	0.660	1.03 (0.80–1.32)	0.831
Good functional outcome^b^	0.81 (0.74–0.90)	**<0.001**	0.82 (0.73–0.93)	**0.002**
Mortality	1.01 (0.90–1.13)	0.853	0.92 (0.80–1.06)	0.239

OR: odds ratio.

In the random effects model treatment center is used as a random effect Age, sex, atrial fibrillation, hypercholesterolemia, hypertension, treatment with IVT prior to EVT, year of treatment and NIHSS score at presentation are used as fixed effects. *p*-values <0.05 were considered significant and are highlighted in bold.

a According to ECASS 2 criteria.

b Defined as a modified Rankin scale score = 0–2, 3 months after stroke onset.

When focusing on the more recent years from 2019 to 2022 in EVA-TRISP, outcomes in the early versus the extended time window were comparable to those observed in the entire time period (years 2016–2022) (Table 2). For patients treated in the extended time window, there were slightly higher rates of good functional outcome, lower NIHSS scores 24 h after EVT and lower incidence of sICH in the 2019–2022 cohort as compared to the 2016–2022 EVA-TRISP cohort.

### Comparison of results with randomized controlled trials

In comparison with both DAWN and DEFUSE 3 treatment arms, patients treated in the extended window in the EVA-TRISP cohort were older (respectively 72.6 vs 69.4 vs 70 years). Patients in EVA-TRISP had a lower median NIHSS score at presentation (13 vs 17 vs 16), and higher rates of patients treated with IVT prior to EVT (22% vs 5% vs 11%). The median time from onset or LSW to groin in EVA-TRISP was 630 min. In DAWN, the median interval between the time that the patient was last known to be well to randomization was 732 min and in DEFUSE 3 the median time from stroke onset to randomization was 653 min (Supplemental File 3).

**Figure 2.**
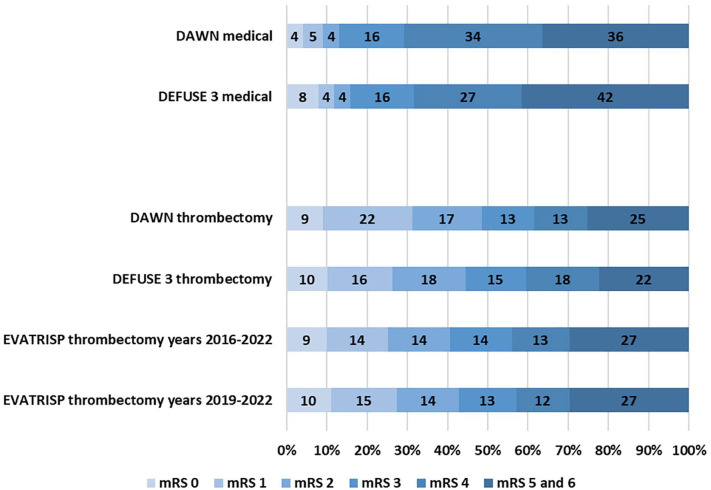
Distribution of Modified Rankin Scale scores after 3 months for EVT patients treated in the extended time window in EVA-TRISP in comparison with clinical trials.

Figure 2 shows the distribution of the unadjusted mRS scores 3 months after stroke onset for the EVA-TRISP cohort and for the DEFUSE 3^6^ and DAWN^7^ trials for patients treated in the extended time window. There were lower rates of favorable outcome (mRS scores between 0 and 2) in EVA-TRISP (37%) in comparison with DAWN (49%) and DEFUSE 3 (44%). When focusing on the higher mRS scores of 5 and 6, there were similar occurrences in EVA-TRISP (27%) and DAWN (25%) and higher occurrences in EVA-TRISP as compared to DEFUSE 3 (22%). Rate of sICH was lower in EVA-TRISP (4.6%) compared to DAWN and DEFUSE 3 (respectively 6% and 7%).

However, when excluding patients with a pre-mRS score higher than 2, the rate of good functional outcome in EVA-TRISP (40.9%) was slightly higher and more in line with the trials (Supplemental File 4).

## Discussion

The findings of this study shed light on outcomes after treatment with EVT in the extended time window in a large real-world cohort. In the EVA-TRISP cohort the proportion of patients undergoing EVT in the extended time window increased over the years. As expected, the proportion of good functional outcome was lower in the group treated in the extended time window in comparison with the early time window. However, sICH and mortality rates were comparable in both time windows. In EVA-TRISP rates of good functional outcome were slightly lower than the DAWN and DEFUSE 3 clinical trials. Nevertheless, results were encouraging as outcomes for EVT treated patients in this real-world cohort were notably better than the patients in the control arms from the trials.

Because a patient’s eligibility for EVT beyond 6 h after onset is often assessed using the inclusion criteria of the DAWN and DEFUSE 3 trials, our cohort results are compared with these trials. Our findings are also in line with results of the patient level meta-analysis of AURORA (Analysis of Pooled Data from Randomized Studies of Thrombectomy More Than 6 hours After Last Known Well),^26^ where smaller clinical trials investigating the effect of treatment with EVT beyond 6 h were also included. In AURORA the rate of good functional outcome in the treatment arm was 46% versus 37% in EVA-TRISP. It should be noted that the AURORA patient pool largely consisted of DAWN and DEFUSE 3 patients. Our results were also comparable with several other larger cohort studies comparing outcomes in early versus extended time windows. A somewhat older study performed within the MR CLEAN^19^ registry, analyzed data collected before the results of the trials (from 2014 till 2017). This study showed a much smaller proportion of patients treated with EVT in the extended window, but rates of good functional outcome (43%) and mortality (24%) after 3 months and sICH (5%) compared to outcomes observed in this study.^19^ In the Trevo registry cohort study,^20^ DAWN and DEFUSE 3 basic demographic and clinical criteria were used to select patients for analysis. Even higher rates of good functional outcome after 3 months were observed in the Trevo DAWN-like (50%) and DEFUSE 3-like (52%) patient groups treated in the extended window as compared to EVA-TRISP. Also, lower rates of mortality and sICH were observed in the DAWN-like and DEFUSE 3-like patients groups treated in the extended window in the Trevo cohort (respectively 11% and 10% vs 21% in EVA-TRISP).^20^ Possibly, the domains of eligibility for EVT were more strictly selected. Another large multi-center cohort study with the Get With The Guidelines-Stroke (GWTG-S) database^22^ also studied outcomes after treatment in early versus extended time window. Although this study did not analyze rates of functional outcome or mortality, rate of sICH observed in the extended window in the GWTG-S (4.8%) cohort was similar to EVA-TRISP (4.6%).^22^

In EVA-TRISP only patients treated with EVT were enrolled, precluding a comparison with untreated patients in the extended window. Many specific and individual reasons for selection for treatment remain unknown. We could also not compare our results with those of DAWN and DEFUSE 3 trials^6,7^ directly, due to limited data on imaging findings in our registry. We could however compare some of the most important clinical baseline variables. DAWN and DEFUSE 3 were also not fully comparable with each other since the treatment window for inclusion differed. Due to the real life nature of this study the mRS score was not evaluated centrally but was assessed either by telephone calls, outpatient visits or electronical/postal questionnaires by the treatment centers. In this cohort we observed a considerable amount of missing values for our outcome variables sICH and mRS score. Since our baseline variables had in contrast high data completeness and imputation techniques for outcome variables are possible but not advised, instead the proportions of missing data were shown in all tables for transparency.

We observed relatively lower rates of successful recanalization in both early and late window treated patients. Apart from the timing of EVT, this might be a significant contributor to the lower rates of good functional outcome in EVA-TRISP since both DAWN and DEFUSE 3 had higher rates of successful recanalization. The clinical trials had stricter inclusion criteria for pre-mRS scores as compared to EVA-TRISP. Patients with a pre-mRS score higher than 1 (DAWN) or 2 (DEFUSE 3) were excluded from the trials while in EVA-TRISP a considerable proportion (10%) of patients with a pre-mRS score higher than 2 received EVT and were included in analysis. In DAWN and DEFUSE 3 only patients with NIHSS scores at presentation of respectively 10 or higher or 6 or higher were included while EVA-TRISP also included patients with lower NIHSS scores. The median NIHSS scores at admission in EVA-TRISP were lower (median 13 [IQR 7–18] as compared to DAWN (17 [13–21])^7^ and DEFUSE 3 (16 [10–20])^6^ (Supplemental File 3). This may have impacted the 90-day mRS scores since patients with good functional outcome could have had milder stroke symptoms at admission. Baseline imaging data in EVA-TRISP were incomplete, precluding meaningful analysis. It is unknown how baseline imaging findings of the patients treated in extended time windows in EVA-TRISP compare to patients treated during clinical trials. While a potential difference in baseline imaging findings might also be a factor contributing to the difference in functional outcome in EVA-TRISP and the trials. EVA-TRISP differed from DAWN and DEFUSE 3 on multiple domains and the domains of these trials also varied between themselves; precluding a meaningful analysis. Therefore, we did not perform a direct comparison for instance with adjusted comparison models or propensity score matching. Despite this variability between EVA-TRISP and the trials, outcomes were encouraging.

One of the strengths of this study was the data completeness for most of the variables, which was remarkable given the pragmatic and observational setting of EVA-TRISP. Other strengths were the large cohort size, the participation of dedicated comprehensive stroke centers from different European countries and the inclusion of patients over a large time period.

## Conclusions

In this large cohort study EVT was a safe and effective treatment option for patients treated in the extended window. Rates of good functional outcome, although lower than those observed in the DAWN and DEFUSE 3 trials, were still remarkably higher than the untreated patients in these trials. While it remains clear that outcomes are time dependent, EVT remains a promising treatment for patients presenting beyond 6 h of symptom onset.

## Supplemental Material

sj-docx-1-eso-10.1177_23969873241277437 – Supplemental material for Endovascular treatment in patients with acute ischemic stroke presenting beyond 6 h after symptom onset: An international multicenter cohort study of the EVA-TRISP collaboration Supplemental material, sj-docx-1-eso-10.1177_23969873241277437 for Endovascular treatment in patients with acute ischemic stroke presenting beyond 6 h after symptom onset: An international multicenter cohort study of the EVA-TRISP collaboration by Nabila Wali, Lotte J Stolze, Leon A. Rinkel, Mirjam R Heldner, Madlaine Müller, Marcel Arnold, Pasquale Mordasini, Jan Gralla, Philipp Baumgartner, Corinne Inauen, Laura P Westphal, Susanne Wegener, Patrik Michel, Simon Trüssel, Laura Mannismäki, Nicolas Martinez-Majander, Sami Curtze, Georg Kägi, Livio Picchetto, Maria Luisa Dell’Acqua, Guido Bigliardi, Christoph Riegler, Christian H Nolte, Miguel Serôdio, Miguel Miranda, João Pedro Marto, Andrea Zini, Stefano Forlivesi, Luana Gentile, Carlo W Cereda, Alessandro Pezzini, Ronen R Leker, Asaf Honig, Ivana Berisavac, Visnja Padjen, Marialuisa Zedde, Laurien S Kuhrij, Renske M Van den Berg-Vos, Stefan T Engelter, Henrik Gensicke and Paul J Nederkoorn in European Stroke Journal
