# Supplementary material for: Endovascular treatment in patients with acute ischemic stroke presenting beyond 6 h after symptom onset: An international multicenter cohort study of the EVA-TRISP collaboration
Source: Eur Stroke J. 2024 Sep 8;10(2):422–30. doi: 10.1177/23969873241277437 (PMC11569508; doi:10.1177/23969873241277437)
Supplement: sj-docx-1-eso-10.1177_23969873241277437 – Supplemental material for Endovascular treatment in patients with acute ischemic stroke presenting beyond 6 h after symptom onset: An international multicenter cohort study of the EVA-TRISP collaboration [file sj-docx-1-eso-10.1177_23969873241277437.docx]

***Supplementary file 1.* *Data collection period per center***

| **Center** | **Period of data collection** | **Number of patients included** |
| --- | --- | --- |
| **St Gallen** | Jan-2016 till Dec-2022 | 660 |
| **Basel** | Jan-2016 till Jul-2022 | 600 |
| **Bern** | Jan-2016 till Sept-2022 | 1542 |
| **Brescia** | Jan-2016 till Jul-2021 | 424 |
| **Lausanne** | 2016 till 2022 | 615 |
| **Zürich** | Jan-2016 till Dec-2022 | 911 |
| **Helsinki** | Jan-2016 till Dec 2020 | 684 |
| **Modena** | Jan-2016 till sept-2022 | 401 |
| **Amsterdam** | Jan-2016 till March-2022 | 998 |
| **Berlin** | 2016-2021 | 607 |
| **Belgrad** | Jan-2018 till Dec 2021 | 204 |
| **Jerusalem** | Jan 2016 - March 2022 | 250 |
| **Lugano** | Jan-2016 till Dec-2022 | 412 |
| **Reggio Emilia** | Oct-2016 till May-2021 | 128 |
| **Bologna** | Sept 2018 till Oct 2021 | 357 |
| **Lisbon** | Jan-2016 till Apr-2022 | 520 |

***Supplementary file 2. Numbers of included and excluded patients in analyses***


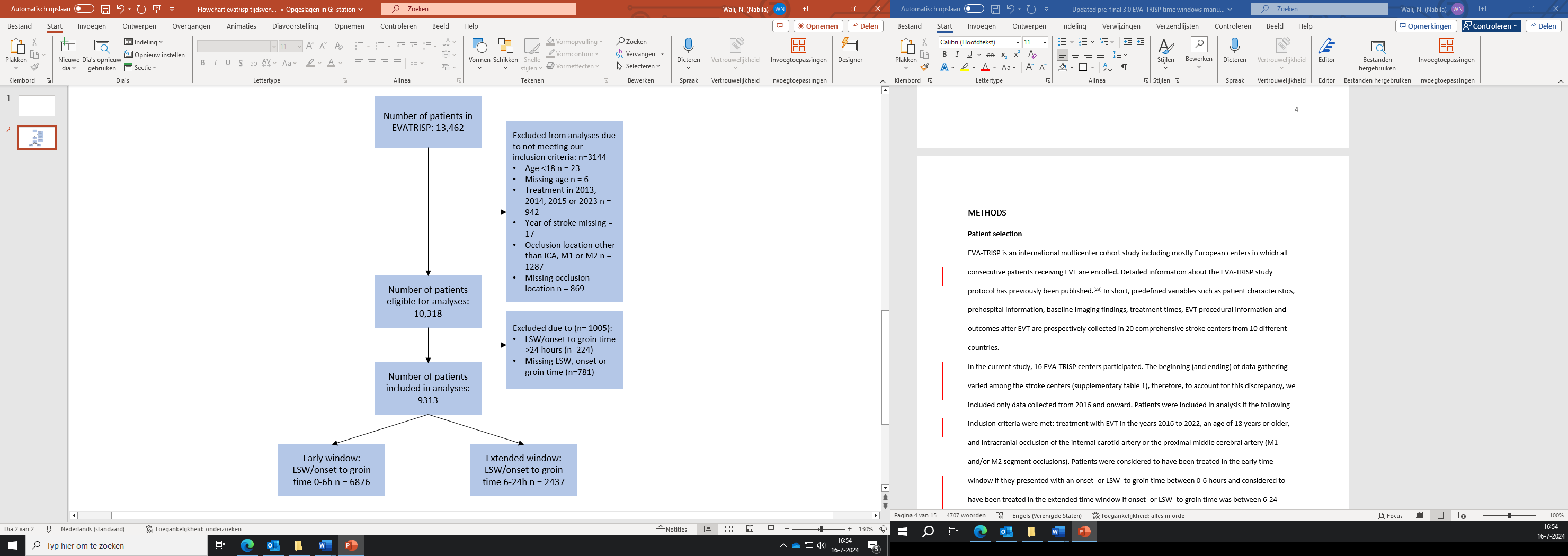


***Supplementary file 3. Baseline characteristics and results of the* DAWN^7^ and DEFUSE 3^6^ *trials***

|  | DAWN trial | | DEFUSE 3 trial | |
| --- | --- | --- | --- | --- |
|  | Thrombectomy (treatment between 6-24hours) | Control | Thrombectomy (treatment between 6-16 hours) | Control |
| Number of patients | 107 | 99 | 92 | 90 |
| Age, mean (SD) and median (IQR) | 69.4 (14.1) | 70.7 (13.2) | 70 (59-79) | 71 (59-80) |
| Female sex, n (%) | 65 (61) | 48 (48) | 46 (50) | 46 (51) |
| Atrial fibrillation, n (%) | 43 (40) | 24 (24) |  |  |
| Diabetes mellitus, n (%) | 26 (24) | 31 (31) |  |  |
| Hypertension, n (%) | 83 (78) | 75 (76) |  |  |
| Previous ischemic stroke of transient ischemic attack, n (%) | 12 (11) | 11 (11) |  |  |
| NIHSS at admission, median (IQR)* | 17 (13-21) | 17 (14-21) | 16 (10-20) | 16 (12-21) |
| Treatment with IVT, n (%) | 5 (5) | 13 (13) | 10 (11) | 8 (9) |
|  |  |  |  |  |
| Grade 2b or 3 on mTICI scale, n (%)** | 90 (84) | NA | 69 (76) | NA |
|  |  |  |  |  |
| Interval between time that patient was last known to be well and randomization in minutes, median (IQR) | 732 (612-978) | 798 (564-948) |  |  |
|  |  |  |  |  |
| Time from first observation of symptoms to randomization in minutes, median (IQR) | 288 (216-372) | 336 (216-468) |  |  |
|  |  |  |  |  |
| Time from stroke onset to randomization in minutes, median (IQR) |  |  | 653 (526-741) | 644 (522-784) |
|  |  |  |  |  |
| **Outcomes** |  |  |  |  |
| mRS scores at 90 days, % |  |  |  |  |
| 0 | 9 | 4 | 10 | 8 |
| 1 | 22 | 5 | 16 | 4 |
| 2 | 17 | 4 | 18 | 4 |
| 3 | 13 | 16 | 15 | 16 |
| 4 | 13 | 34 | 18 | 27 |
| 5 | 25 | 36 | 8 | 16 |
| 6 |  |  | 14 | 26 |
| Functional independence at 90 days (mRS 0-2), n (%)+ | 52 (49) | 13 (13) | 41 (45) | 15 (17) |
| Stroke-related death at 90 days, (%) | 17 (16) | 18 (18) | 13 (14) | 23 (26) |
| Death from any cause at 90 days, (%) | 20 (19) | 18 (18) |  |  |
| Symptomatic intracranial hemorrhage^ | 6 (6) | 3 (3) | 6 (7) | 4 (4) |

*SD = standard deviation, NIHSS = National Institute of Health Stroke Scale, IQR = interquartile range, IVT = intravenous thrombolysis*

**Scores on the National Institutes of Health Stroke Scale (NIHSS) range from 0 to 42*

**The modified Thrombolysis in Cerebral Infarction (mTICI) scale ranges from 0 to 3, with a grade of 2b or 3 indicating reperfusion of more than 50% of the affected territory.

+Functional independence was defined as a score of 0, 1, or 2 on the modified Rankin scale

^In the DAWN trial symptomatic intracranial hemorrhage (sICH) was defined according to European Cooperative Acute Stroke Study III criteria as the presence of extravascular blood in the cranium that was associated with an increase in the NIHSS score of 4 points or more or death and was judged to be the predominant cause of neurologic deterioration within 24 hours after randomization.

In the DEFUSE 3 symptomatic intracranial hemorrhage was defined as an increase of at least 4 points in the NIHSS score that was associated with brain hemorrhage on imaging within 36 hours after symptom onset. In DEFUSE 3 among the patients with symptomatic intracranial hemorrhage, the hemorrhage was rated as parenchymal hematoma type 2 (dense blood clot exceeding 30% of the infarct volume with substantial space-occupying effect; in two patients in the endovascular-therapy group and three patients in the medical-therapy group), parenchymal hematoma type 1 (blood clot not exceeding 30% of the infarcted area with some mild space-occupying effect; in one patient in the endovascular-therapy group), hemorrhagic infarction type 2 (confluent petechiae within the infarcted area, but without space-occupying effect; in three patients in the endovascular-therapy group), or hemorrhagic infarction type 1 (small petechiae along the margins of the infarct; in one patient in the medical-therapy group).

***Supplementary file 4.*** *Outcomes after EVT per treatment window for the years 2016-2022 and the years 2019-2022. Patients with pre-mRS scores >2 are excluded.*

|  | Years 2016-2022 | | | Years 2019-2022 | | |
| --- | --- | --- | --- | --- | --- | --- |
|  | Early time window | Extended time window | p-value | Early time window | Extended time window | p-value |
| Number of patients | 5826 | 2076 |  | 3399 | 1426 |  |
| NIHSS after 24 hours, median (IQR) | 8 (3-16) | 9 (4-16) | **<0.001** | 7 (3-15) | 9 (4-16) | **<0.001** |
| Missing, n (%) | 650 (11.2) | 173 (8.3) |  | 336 (9.9) | 134 (9.4) |  |
| ∆ NIHSS, mean (SD) | -3.8 (8.1) | -1.8 (7.5) | **<0.001** | -4.0 (7.8) | -2.0 (7.5) | **<0.001** |
| Missing, n (%) | 669 (11.5) | 185 (8.9) |  | 346 (10.2) | 141 (9.9) |  |
| Symptomatic ICH*, n (%) | 264 (4.5) | 104 (5.0) | 0.919 | 147 (4.3) | 65 (4.6) | 0.948 |
| Missing, n (%) | 1130 (19.4) | 247 (11.9) |  | 561 (16.5) | 183 (12.8) |  |
| mRS score after 3 months, (%) |  |  | **<0.001** |  |  | **<0.001** |
| 0 | 13.7 | 9.9 |  | 14.9 | 10.7 |  |
| 1 | 17.4 | 15.6 |  | 17.7 | 16.1 |  |
| 2 | 15.7 | 15.5 |  | 14.4 | 15.6 |  |
| 3 | 13.4 | 14.3 |  | 12.4 | 13.5 |  |
| 4 | 10.6 | 13.5 |  | 10.4 | 12.3 |  |
| 5 | 4.1 | 5.6 |  | 3.9 | 5.4 |  |
| 6 | 17.5 | 18.1 |  | 16.3 | 17.5 |  |
| Missing, n (%) | 438 (7.5) | 158 (7.6) |  | 337 (9.9) | 125 (8.8) |  |
| Good functional outcome+, n (%) | 2729 (46.8) | 850 (40.9) | <0.001 | 1599 (47.0) | 605 (42.4) | **<0.001** |
| Mortality^, n (%) | 1018 (17.5) | 376 (18.1) | 0.497 | 554 (16.3) | 250 (17.5) | 0.381 |

*NIHSS = National Institutes of Health Stroke Scale, IQR = interquartile range, SD = standard deviation, mRS = modified Rankin scale*

*∆ NIHSS after 24 hours minus NIHSS at presentation*

** According to ECASS II criteria*

*+Defined as a modified Rankin scale score = 0-2,* three months after stroke onset

*^Defined as a mRS score = 6, three months after stroke onset*
